# Unsupervised query reduction for efficient yet effective news background linking

**DOI:** 10.7717/peerj-cs.1191

**Published:** 2023-01-13

**Authors:** Marwa Essam, Tamer Elsayed

**Affiliations:** Qatar University, Doha, Qatar

**Keywords:** News linking, News recommendation, Keyword extraction, *Ad-hoc* retrieval, Query reduction, Efficiency analysis

## Abstract

In this article, we study efficient techniques to tackle the news background linking problem, in which an online reader seeks background knowledge about a given article to better understand its context. Recently, this problem attracted many researchers, especially in the Text Retrieval Conference (TREC) community. Surprisingly, the most effective method to date uses the entire input news article as a search query in an *ad-hoc* retrieval approach to retrieve the background links. In a scenario where the lookup for background links is performed online, this method becomes inefficient, especially if the search scope is big such as the Web, due to the relatively long generated query, which results in a long response time. In this work, we evaluate different unsupervised approaches for reducing the input news article to a much shorter, hence efficient, search query, while maintaining the retrieval effectiveness. We conducted several experiments using the Washington Post dataset, released specifically for the news background linking problem. Our results show that a simple statistical analysis of the article using a recent keyword extraction technique reaches an average of 6.2× speedup in query response time over the full article approach, with no significant difference in effectiveness. Moreover, we found that further reduction of the search terms can be achieved by eliminating relatively low TF-IDF values from the search queries, yielding even more efficient retrieval of 13.3× speedup, while still maintaining the retrieval effectiveness. This makes our approach more suitable for practical online scenarios. Our study is the first to address the efficiency of news background linking systems. We, therefore, release our source code to promote research in that direction.

## Introduction

In today’s digital era, many people follow online news portals or digital media to learn about different subjects, events, or topics. Being at almost no cost, with 24/7 updates on the latest news around the globe, online news articles have become one of the most vital sources of knowledge. However, as opposed to other long-text knowledge sources, such as books or research articles, news articles are often limited in length. As a result, a single news article is rarely self-contained with all the information about the topic it discusses. In many times, a reader of a news article finds himself or herself in need of some in-depth background information to understand or conceptualize the article he or she is reading. In fact, a qualitative analysis of online news readers engagement in news websites (O’Brien, 2011) showed that readers of news articles often need more depth in the articles to situate the news stories in their proper temporal context. Another recent study Lehmann et al. (2017) showed that many readers, engage in a story-focused session, searching for information related to a news story, and if this information is not entirely found in one article, as expected, they tend to use search engines to find other resources related to the same story.

To automate the knowledge acquisition process for readers, links to other resources that provide the required background knowledge on a news article may be added to the article’s content (O’Brien, 2011). While news articles in almost all online news providers have already links to other articles, they do not always link to useful background or related context; instead, they often link to other articles written by the same author, articles that are most-viewed by the readers, or top headlines of the day. *Background links*, if they exist, are added manually by the news author to other articles that he or she thinks might be related to the article’s topic or story. Aside from the need to automate this process, readers may require information that is not found in the articles linked by the author, such as different viewpoints on the article’s subject, or an expanded scope on a specific subtopic. Furthermore, due to competition and to keep readers as long as possible on their websites, news providers rarely link articles to external sources. However, external resources to the news provider (*e.g*., Wikipedia pages, articles from other news providers, *etc*.) might also be useful to the reader.

In 2018, the Text REtrieval Conference (TREC) initiated a news background linking task to address the *news background linking problem*, for which systems are built to take an input article and return a list of links to other resources that provide background and contextual knowledge on the given article (Soboroff, Huang & Harman, 2018). For this task, TREC released a dataset of about 600k news articles, from the Washington Post newspaper, to be the source collection. While many research teams participated in the task, surprisingly the most effective method reported to date adopted a simple *ad-hoc* search approach, where the full content of the input article was used as a search query to retrieve the background links from an indexed collection. This method, however, is inefficient, specifically for relatively long articles. This is because, regardless of the model used for scoring the candidate articles, inverted lists of all terms of this article are typically processed to determine the ranking of the retrieved articles (Moffat et al., 2007). As expected, with the large number of terms in the input article, the pool of candidate articles increases, even with pruning strategies (Macdonald, Tonellotto & Ounis, 2012). Furthermore, if we consider the online scenario when a reader of a news article requests background links from the Web, this method will be even more costly due to the size of the Web, the nature of its growth, and the collection of documents that makes it up.

To overcome the above limitation when addressing the problem, in this article, we aim to study unsupervised approaches that can efficiently retrieve the background links required for an input article, while maintaining the same (or comparable) effectiveness reported so far by using the full input article as a search query. To our knowledge, this is the first study that addresses the efficiency issue in the domain of news background linking problem; all other related studies focused on the effective retrieval of the background links, regardless of the time taken to obtain those links. To achieve our goal, we hypothesize that selecting a much fewer number of terms from the input article, instead of its full content, might be enough to create an effective search query for our task. In other words, we aim to reduce the long query article to a much shorter search query while maintaining its effectiveness.

While *query reduction* was studied before in literature (Kumaran & Carvalho, 2009; Balasubramanian, Kumaran & Carvalho, 2010), most of that work assumed that the long queries are a description of the user’s information need in Web search, which is maximum of 30 terms in length. Therefore, previous work considered techniques for assessing short sub-queries (2 to 6 terms), which in many cases typically involve post-retrieval features. This is, indeed, infeasible to apply for the lengthy news articles that have hundreds of terms, as the number of sub-queries to be assessed will pose even more time for retrieval compared to the full article, which contradicts our aim from the reduction.

To achieve our goal, in this article, we aim to address the following research questions:

1. **RQ1:** Can we effectively retrieve the background links by just using the *lead paragraphs* of the input article to construct the search query?
2. **RQ2:** How effective and efficient are *typical keyword extractions* techniques for this task?
3. **RQ3:** Which query reduction technique is more effective if we allow further reranking of the candidate links?

We designed a number of experiments to answer the above questions. We first explored the idea of using only the lead paragraphs of the news article as the reduced search query. The results show that it considerably reduces the retrieval effectiveness, and that most of the article’s content has be considered to achieve a relatively good retrieval effectiveness. We next experimented with the state of the art unsupervised keyword extraction techniques for constructing a weighted search query out of the input news article. Our results show that using *Yake* (Campos et al., 2020), a recently introduced statistical keyword extraction technique, yields an effectiveness that is not significantly different from the state-of-the-art background linking technique (which uses the full input article as a query), while being significantly more efficient in retrieval. Moreover, we found that we can adopt the simple traditional *TF*-*IDF* weighting mechanism to further omit “unimportant” search terms from the search queries generated by *Yake* for a further speedup while maintaining the effectiveness. We further evaluated the ability of the keyword extraction techniques to construct search queries that are able to retrieve as many as possible of relevant background links (*i.e*., promising for further reranking). Our results show that graph-based keyword extraction methods, such as *k*-Core (Rousseau & Vazirgiannis, 2015) and *k*-Truss (Tixier, Malliaros & Vazirgiannis, 2016), have the potential for achieving the highest effectiveness after the reranking of the initially-retrieved articles.

Our contribution in this article is four-fold:

- While many researchers addressed the background linking problem, mainly within the scope of TREC news track, our study is the first to highlight the efficiency aspect of this problem, aiming to build an efficient background linking system that maintains the far most obtained effectiveness, besides being the first to extensively review the literature for this problem, considering as well other resources for background linking than news articles.
- We present a new comparative study between several state-of-the-art unsupervised keyword extraction techniques for a new downstream task (news background linking) in which they were never evaluated, in terms of both effectiveness and efficiency.
- We show that we can quite efficiently reduce the query response time needed for the retrieval of the background links, while maintaining the retrieval effectiveness of the full article approach, using simple unsupervised statistical keyword extraction techniques.
- We make our source code for running the different methods and experiments publicly available.

The rest of the article is organized as follows. An extensive literature review of linking news articles to external sources of information for contextualization purposes is presented first, followed by a definition of our research problem and how we distinguish it from other related problems. Subsequently, we detail our proposed query reduction approach, including a summarized description of the keyword extraction techniques that we experimented with. Finally, results of our experiments are discussed and conclusions are presented.

## Related work

In this section, we review the work done for news articles contextualization; *i.e*., to allow readers to contextualize and understand better the content of news articles. We start by reviewing the work done mostly in TREC for news background linking, which considers other news articles than the query article as sources of knowledge. We then review the work that was conducted earlier and considered other sources of background knowledge (such as Wikipedia pages and scientific articles among others).

### News articles as sources of background knowledge

A news background linking task was recently introduced as a new challenge in the news track in TREC 2018 (Soboroff, Huang & Harman, 2018), and as a follow-up task in TREC 2019 (Soboroff, Huang & Harman, 2019), and TREC 2020 (Soboroff, Huang & Harman, 2020). A number of teams participated in this challenge proposing different methods that are surveyed below. It is important to highlight that all the methods surveyed in this section did not achieve an effectiveness higher or even equal to the method that uses simply the whole query article as a search query to retrieve the background links. Accordingly, we focused in our experiments on the comparison with solely this later method, as we will discuss later in the Experimental Evaluation Section.

#### Ad-hoc based retrieval of background links

Some proposed methods addressed the task using an *ad-hoc* search approach, in which an input query article is analyzed to construct a search query. Yang & Lin (2018) introduced a number of methods that were all implemented in Anserini (Yang, Fang & Lin, 2017). The proposed methods selected the query terms as the 1,000 article terms having the highest *TF*-*IDF* values, or as the first 1,000 terms that appeared in either the article or some selected paragraphs. Since the average length of the articles in the Washington Post collection was around 400 terms (after removing stop words), we can say that this method, for many of the articles, used the full article as a search query. Lopez-Ubeda et al. (2018) constructed different queries using the article’s title, the full body text, and both the title and the body, to generate initial results. Candidate articles were then selected based on a k-means clustering to diversify the results to the news reader.

In an attempt to find the most influential terms to be used in the search query, Bimantara et al. (2018) selected the key phrases extracted using *TextRank* (Mihalcea & Tarau, 2004) (a basic graph-based text analysis algorithm), and used these keyphrases as the search query along with a list of named entities extracted also from the query article. Documents were also re-ranked considering their publication dates to allow articles that were published close to the input article to appear at the top of the retrieved list. Essam & Elsayed (2019) also used graph-based keyword extraction mechanisms to determine the most influential terms from the query article, and used up to 20% of the extracted terms from the query article as a search query.

Forming multiple search queries from the input article, Lua & Fang (2018) suggested splitting an article into paragraphs and extracting query terms from each paragraph to form the search queries using a probabilistic model. Before indexing, the entities in the text were identified using DBpedia Spotlight (Daiber et al., 2013), and they were subsequently replaced by their canonical forms (Lu & Fang, 2019). In 2019, the authors suggested, additionally, to assign different weights to the spotted DBpedia entities in the input document based on its surrounding context. To do so, they combine the words before and after each occurrence of each entity, and generate a language model based on the words. They then compute the KL-divergence between this context language model and the document language model to find the entity weight. The probabilistic weight distribution of the regular words and of the entities of the query article is then used in the retrieval process of background links.

Exploring relevance feedback and query expansion, Missaoui et al. (2019) initiated a search first using the named entities extracted from the query article’s title. They then used the top 10 retrieved articles in expansion to update the search query. Ding et al. (2019) also adopted similar approach. They did not use only named entities though for their initial query; instead, they used the title and body of the input article with weights equal to 0.3 and 0.7 respectively. They also assumed that the top 10 retrieved articles are relevant and the lower 10 retrieved documents from a 100 set are non-relevant. For retrieval, they experimented with BM25 and Query Likelihood models, and for expansion, they studied Rocchio and RM3.

#### Supervised retrieval of background links

To learn a model that estimates the usefulness of a background article to a query article, Foley, Montoly & Pena (2019) constructed different linear learning to rank retrieval models trained on the 2018 collection. To select the best set of features for the model, the Coordinate Ascent model (Metzler & Croft, 2007) was adopted. Similarly, Qu & Wang (2019) trained a multi-class classifier to re-rank background documents using logistic regression. The classifier classifies the candidate articles from 0 to 4 according to how much knowledge they provide to the query article. To produce the background documents, the top 80 frequent words (using *TF*-*IDF* scores) were used as a query to retrieve an initial set of 1,000 candidate background documents. This set is then re-ranked by the classifier. Khloponin & Kosseim (2019) suggested representing all documents in the collection as vectors using Doc2Vec distributed representation (Le & Mikolov, 2014). Based on the assumption that documents that have some background knowledge to the query document are closer in the vector space, they then computed the cosine similarity between the query document and all other documents in the collection. Similar to this work, Day, Worley & Allison (2020) computed embeddings for the documents using Sentence-BERT. Precisely, an initial set of background links were first retireved using the highest in *TF*-*IDF* terms as a search query, then for each document in this set, an embedding is generated by pooling the embedding vectors of the leading three paragraphs generated by Sentence-BERT. Finally, the cosine similarity between the embeddings of both the query and each of the candidate articles was used to generate the final rank of background links. Khloponin & Kosseim (2021) further explored a number of document embedding representations, with different proximity measures, and found that GPT2 and XLNet embeddings generally lead to higher performance in the semantic representation of articles for this task.

Computing their own embedding vectors for the dataset, Gautam & Mandar Mitra (2020) trained a word2vec model on all articles, where each word in the *corpus* was represented as a 300 dimensional vector, and an article was represented as the sum of the vectors corresponding to the top 25 words contained in the article. To retrieve the background links, the authors initially retrieved a set of articles using a search query composed of the 80 highest terms in *TF*-*IDF* values. Documents were then reranked given thier retrieval score and the cosine similarity between its embeddings and the query article embeddings. Deshmukh & Sethi (2020) also computed their own embedding representation of the query and candidate documents, by first extracting important keywords from the text, then treating the concatenation of these keywords as a sentence that is fed to Sentence-BERT to obtain the required embeddings.

Ak et al. (2020) suggested further two supervised based methods for background linking. The first used BERT-based extractive summarization methods to generate a summary of 180 words for the news article, then used this summary as a search query. The second fine-tuned next sentence prediction task in BERT for background linking. Since BERT input is limited to 512 tokens, both the query article and the candidate background article were cropped to half of 512. In an attempt to analyze the different topics discussed in the news articles and how much the common topics between articles affect relevance in this problem, Ajnadkar et al. (2021) used a trained LDA model to analyze the news articles in the Washington Post dataset and obtain its topic distributions, then find the most similar articles to a given query article based on the maximum cosine similarity with its topics.

### Other sources of background knowledge

Aside from the work that was proposed for the background linking task in TREC, there has been some research in contextualizing the news articles using external sources (*i.e*., other than articles from the same source). Ravenscroft, Clare & Liakata (2018) built a system that links a news article that reports or discusses a specific scientific research article to this mentioned article. The content of each news article was processed to extract names of authors and scientific organizations. Pairs of author names and organizations were then used as search queries, using Microsoft Academic Knowledge, Scopus, and Springer APIs, to retrieve an initial set of candidate scientific articles. Candidate articles were then scored using a mechanism that is based on the Levenshtein Ratio between the entity mentions in the news article and the author names and affiliations in the scientific work.

To enrich news articles with data visualizations that are available on the Web (*e.g*., maps, line graphs, and bar charts), Lin et al. (2018) proposed a system that uses a learning to rank classifier to link news articles to visualization images that exist in Wikipedia. The candidate images were extracted from the Web pages of named entities that were mentioned in the articles. To train the classifier, features such as the semantic relatedness between the article’s title and content and the image caption or its contained page were used.

Linking the named entities that are mentioned, generally in any text and specifically in news articles, to entities in knowledge sources, has been an active research area in text contextualization. In fact, there is a research problem, denoted as *Wikification*, in which entities are supposed to be spotted in a text then linked back to Wikipedia pages. Recently, a number of systems were proposed for both entity recognition and entity linking, *e.g*., Spotlight (Daiber et al., 2013), Conerel (Phan & Sun, 2018) and Recognyze (Weichselbraun, Kuntschik & Braşoveanu, 2018). To provide mappings between news articles and *Wikidata* events, Rudnik et al. (2019) proposed to check if an article’s publishing date is the same as one of the Wikidata events, if the article mentions the event location in its lead paragraphs, and if the lead paragraphs of the article have high similarity with the Wikidata event’s type and title. For annotating possible events within the articles, the authors used a predefined vocabulary of news events. Linking the news articles content to entities in knowledge sources allows readers to gain more knowledge on the article’s content, and accordingly help the readers contextualize it. Nonetheless, as some articles may not have named entities, additional techniques for contextualization are required. Moreover, the Wikipedia pages or the linked knowledge sources may not contain the up to date information that the reader seeks. Furthermore, we need a general method that allows the retrieval of the required background and context knowledge from any text document regardless of its type or structure. That clearly makes the news background linking problem different from the Wikification problem.

## Background linking problem

In this section, we first formally define the news background linking problem, then we highlight the differences between this problem and other problems that were addressed in literature and aimed generally to find related articles.

### Definition

**Figure 1:**
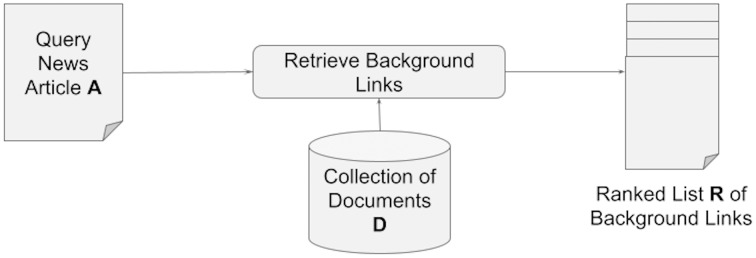
Retrieving a set of background links for a query article.

We define the problem of news background linking as follows: Assuming an input query article *A*, that a reader does not fully comprehend, and given a collection of documents *D*, suggest a ranked list of documents $R \subset D$ that a reader of *A* can read next to provide him or her with the context and background knowledge required to comprehend the content of the query article *A* (see Fig. 1).

**Figure 2:**
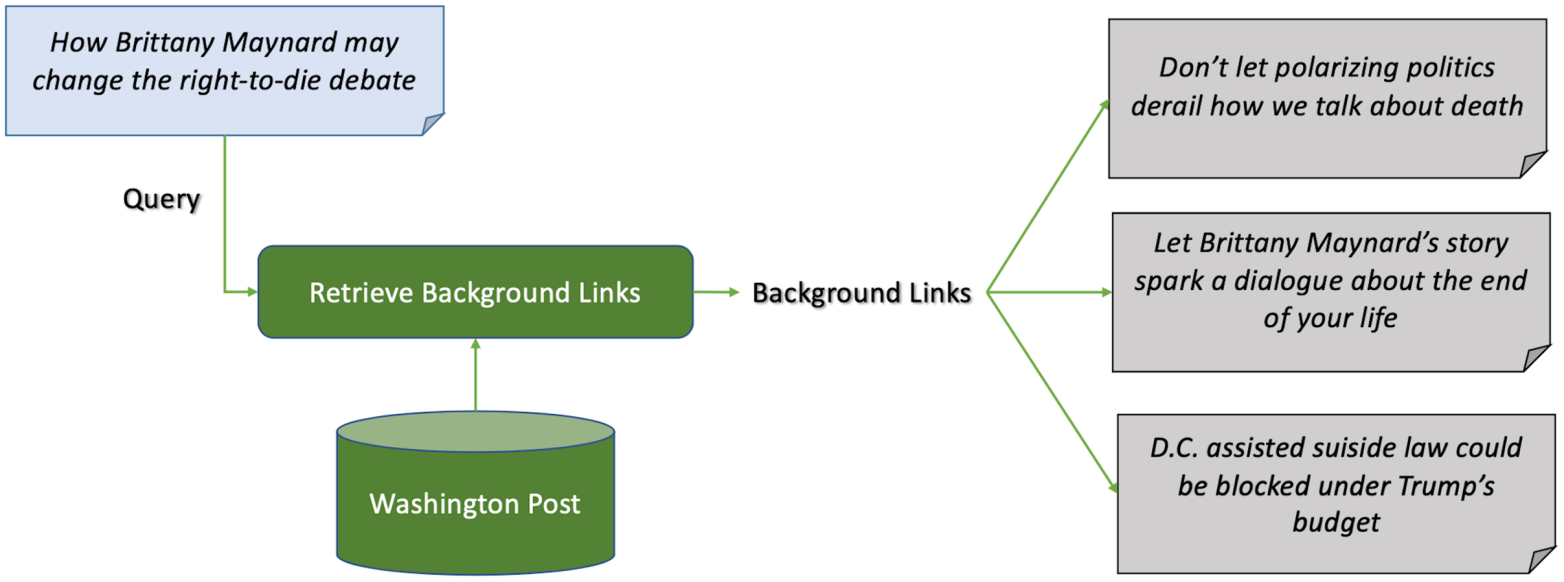
Example 1 of news background linking drawn from the dataset in TREC news track.

**Figure 3:**
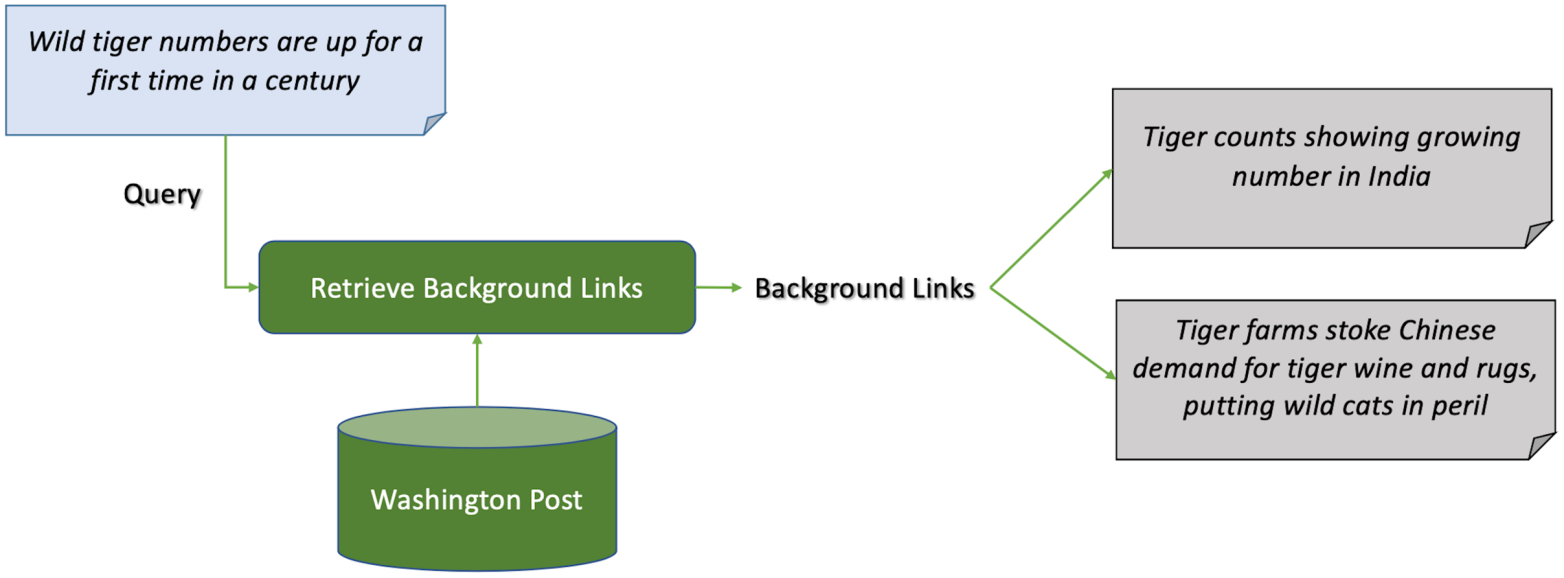
Example 2 of news background linking drawn from the dataset in TREC news track.

Figure 2 shows an example of news background linking, where the query article discusses the effects of the death of a young lady, who took pills to die after being diagnosed with cancer, on the right-to-die debate in the United States. The background articles give the reader more context on the the query article. One article for example discusses a lawsuit in California to approve the right-to-die law. Another discusses the young lady’s campaign before dying, and the third discusses an economical view on approving the right-to-die law in president Trump’s budget. Another example is shown in Fig. 3, where the query article is discussing a recent report on the worldwide increase in the number of tigers. The first background article discusses the case in India and how the government took measures to insure the safety of the tigers, and the other one discusses the contrary effect of having tigers farms in China on their numbers.

### Relation to other problems

A whole body of work has been proposed to deal with finding related articles in general. Some research for instance aimed at recommending articles to the reader that might be of interest to her (Chakraborty et al., 2019). Most often, in this line of research, the proposed system assumed somehow an access to the reader’s information such as her profile, her past online interactions, browsing session or her location, and this information helped in recommending news to her (Chakraborty et al., 2022; Hu et al., 2020). In news background linking, however, the focus is solely on the query article with no access to any user information, *i.e*., the recommended background links are *not personalized*. Furthermore, in news recommendation, the recommended articles are not required for contextualization purposes of the current article. For example, for the query article in Fig. 3, some recommended articles might be about saving rhinos. While they are somehow related and might be of interest to the reader, they are not background and they do not help the reader contextualize the problem of decreasing tiger numbers.

Another close problem is to find clusters of related news articles such that a cluster has a high internal coherence (*i.e*., having articles from the same topic or word distribution), but different from other clusters (Bisandu, Prasad & Liman, 2018; Salih & Jacksi, 2020; Khan et al., 2018). While articles within the cluster might be candidate background to each other, the cluster size is big, and selecting specific articles from the cluster to present to the reader of a specific query article is again a challenging problem. Furthermore, articles that have the same exact information will end up in the same cluster, making them not background to each other.

Finally, there has been some work recently on detecting events within new articles that discuss incidents or stories, and following those story lines along the published articles (Nicholls & Bright, 2019; Naskar et al., 2019; Qian et al., 2019; Örs, Yeniterzi & Yeniterzi, 2020; Conrad & Bender, 2016). While articles published by the same news provider for a specific event may be useful as background to a query article published after them, in some cases, the query article itself might not at all discuss or be triggered by the occurrence of a specific event. It may be a feature article discussing a general topic, *e.g*., an article discussing the increase of college fees and its effects on the education process in the country. For such articles, event or story detection will not help (Essam & Elsayed, 2020). Furthermore, in some cases, an article that does not mention the event of the query article might be a strong background article that helps the reader contextualize the event. Consider, for example, the case for the query in Fig. 2, which shows two very important background articles that do not mention the specific event of the young lady’s death in the query. Furthermore, if an article discusses the exact same event but provides no more added information about that event to the reader (*e.g*., discussing the same event published by another news provider), then this article should not be qualified as background.

## Methodology

As illustrated in the literature, the most common approach to date to address the news linking problem follows an *ad-hoc* retrieval approach. In this approach, the query article *A* is analyzed to produce a search query Q, which is issued against the collection index to retrieve the required background links. The simplest and most effective reported method to date constructs *Q* as a concatenation of the article’s title and its full content. This method is clearly inefficient, though, as we outlined earlier, since the retrieval query includes all the terms of the article, even if they are noisy or uninformative. Therefore, in this work, we assume that a search query that captures only the informative terms can be (at least) as effective as the full article query, yet much more efficient. Moreover, since terms in the article can have different impact in representing the different background aspects of the input article, we assume that constructing a weighted search query, where each unique search term has a real-valued weight, that can be different from other terms, will be helpful in improving the retrieval effectiveness. In fact, adding weighted terms to queries has been shown previously to increase the retrieval effectiveness, specially with the increase in the number of search terms (Qiu & Frei, 1993). In this section, we first describe how we formulate a weighted search query and show how we score the background articles given that query. We then briefly describe the different keyword extraction methods that we chose to adopt in extracting and weighting the search query terms from the input article. Finally, we present a complexity analysis for the adopted methods to contrast their efficiency in extracting the search terms.

### Query formulation and document scoring

Given a query article *A*, we formulate a search query *Q* as follows. Let *n_A_* be the set of unique terms in *A*. We apply a keyword extraction algorithm to assign a weight *w_t,Q_* for each term *t* ∈ *n_A_*. We then choose terms *k* < |*n_A_*| with the highest weights to construct the search query *Q* as follows:

(1) $${Q = \{ (}{{\rm t}_1},\,{w_{{t_1}}},Q),\,{\rm (}{{\rm t}_2},\,{w_{{t_2}}},Q),...,\,{\rm (}{{\rm t}_k},\,{w_{{t_k}}},Q)\}$$

Given *Q*, a document can be scored as follows:

(2) $$score(d,Q) = f(\{ (t,{w_{t,Q}},{w_{t,d}}\;|\;t \in Q \cap d)\} )$$

where $w_{t,d}$ is the weight of term $t$ in $d$ according to the retrieval model $f$ (*e.g*., the term frequency of $t$ in $d$). Finally, the documents with the highest scores can be retrieved as background links for the article *A*.

### Unsupervised keyword extraction techniques

The most significant choice to make when applying our proposed methodology is the keyword extraction technique. We reviewed recent studies that addressed the problem of keyword extraction, focusing on those that compared the performance of state of the art techniques on the gold-standard keyword extraction datasets (Sarwar, Noor & Miah, 2022; Piskorski et al., 2021; Miah et al., 2021; Papagiannopoulou & Tsoumakas, 2020). We also checked the methods that were reported by the recent techniques as effective baselines. Accordingly, we made our selection of the techniques based on the following criteria:

- **Unsupervised methods:** Since the problem we address is very recent, and there is no labeled data for supervised learning, (*i.e*., there is no golden set of keywords extracted from the query articles that can be used to retrieve the best set of background links), we focus only on selecting keyword extraction techniques that are mainly unsupervised.
- **Effective number of keywords:** Our goal from extracting the keywords is to use them to form search queries to retrieve candidate background documents from a big news articles collection. Hence, we prefer the keyword extraction technique that can provide a large number of good representative keywords. In our preliminary experiments, using few search keywords in a retrieval query, even of good quality or representation of the query topic, considerably lowered the retrieval effectiveness. Accordingly, techniques that failed to provide large number of good keywords (30 in our preliminary experiments) were excluded, such as **Teket** (Rabby et al., 2020).
- **Effectiveness on news articles**: When reporting its effectiveness, many keyword extraction studies conduct the experiments on scientific article datasets or even books; however, news articles have special features. They are shorter, less cohesive, and they may discuss multiple subtopics. Hence, we selected the recent techniques that worked best when tested on *English news articles* datasets.

Given the above criteria, we experimented with the techniques listed in Table 1 along with their types. We note that most of the methods are graph-based (*i.e*., they construct a graph of terms from the input document, then analyze it to extract the required keywords). We further experimented with two simple and standard keyword extraction methods, which are Term Frequency (*TF*) and Term Frequency-Inverse Document Frequency (*TF*-*IDF*), as additional baselines to compare against.

**Table 1:** The keyword extraction methods we studied in this work.

Method	Type
*TF*	Statistical
*TF*-*IDF*	Statistical
*k*-Core (Rousseau & Vazirgiannis, 2015)	Graph-based
*k*-Truss (Tixier, Malliaros & Vazirgiannis, 2016)	Graph-based
*PositionRank* (Florescu & Caragea, 2017)	Graph-based
*TopicRank* (Bougouin, Boudin & Daille, 2013)	Graph-based
*MPR* (Boudin, 2018)	Graph-based
*sCake* (Duari & Bhatnagar, 2019)	Graph-based
*Yake* (Campos et al., 2020)	Statistical

A brief description of the evaluated methods and how they weigh the terms is given below. Since we need boosting weights for individual terms to construct search queries, we further show how, for some methods, we computed those boosts given the weights assigned for the extracted keyphrases.

### *k*- Core

*k*-Core (Rousseau & Vazirgiannis, 2015) is a graph-based keyword extraction method that depends on the construction of a graph-of-words for the document being processed, and analyzing this graph to extract the required keywords. Nodes in the graph-of-words are simply the unique words in the text after pre-processing (*e.g*., stop words removal), and edges are added by sliding a window over the text, creating an edge between words that co-occur within this sliding window. In general, the underlying assumption of creating graphs from text and analyzing it is that terms co-occurring within a relatively small window of text have some kind of semantic relatedness regardless of their roles in a sentence, and that this relationship influences the importance of each single term within the text document, leading to better document analysis. To analyze the constructed graph of words, *k*-Core decomposes the graph into a hierarchy of nested subgraphs using graph degeneracy methods, and goes down this hierarchy to identify nodes that are at the core of the graph. This is based on the assumption that nodes in the core are reachable through the graph by many other nodes, making them influential. *k*-Core decomposition relies on peeling away weakly connected nodes to gradually get the core of the graph. The *k*-Core of a graph is the maximal subgraph such that every node has a degree at least *k*, where the degree of a node is the sum of the weights on its connecting edges. Starting with the initial graph and with *k* = 1, the subgraphs are iteratively created with increasing *k* and pruning nodes, and the algorithm stops when reaching *k*-max which is the maximum subgraph that can be created (*i.e*., the subgraph after which it becomes empty).

Assuming that the graph was decomposed into cores, each node is then assigned the core number of the maximum subgraph at which it resides. For keywords extraction, nodes in the *k*-max subgraph or nodes in the lower level of the hierarchy of subgraphs can be marked as the most influential, and used as keywords (Rousseau & Vazirgiannis, 2015). However, in this article, we adopt the work done by Tixier, Malliaros & Vazirgiannis (2016), which assigns a final score to a node *n_i_* as the sum of the core numbers of its neighbors in the original graph. This scoring aims at decreasing the granularity in nodes selection. As suggested, for keyword extraction, if a score is assigned based only on the node’s own core/truss number, then many nodes will end up having the same weight, increasing the number of selected nodes (Bae & Kim, 2014).

### *k*- Truss

*k*-Truss is another graph decomposition method (Tixier, Malliaros & Vazirgiannis, 2016) that is triangle-based, and is based on a cohesive social and communication pattern in graphs introduced by Cohen (2008). A triangle in a graph *G* is the set of three nodes *a, b *and* c* that are pairwise-connected. Instead of pruning weak nodes in the decomposition process as in *k*-Core, *k*-Truss peels weak edges first, then removes nodes with no more connecting edges in the resulting subgraph. Precisely, *k*-Truss prunes an edge from the *k*−1 subgraph if it is not supported by at least *k*−2 other edges that form triangles with that edge. Similar to *k*-Core, we follow the same scoring method to compute the node scores given their truss numbers.

#### PositionRank

*PositionRank* is a also a graph-based keyword extraction algorithm (Florescu & Caragea, 2017) that uses the graph-of-words structure. However, after constructing the graph, *PositionRank* adapts the *PageRank* algorithm (Page et al., 1999), a well known iterative random walk-based algorithm for ranking web pages, to assign scores to nodes (words) in the graph that indicate its importance. Aside from *PageRank* that assigns equal initial scores/weights to all nodes in the graph, *PositionRank* assigns initial scores to nodes (words in this case) based on their positions in the text being analyzed. Precisely, it favors more nodes that occur at the lead of the text, based on the assumption that keywords occur frequently very close to the beginning of a document. In constructing the graph of words, *PositionRank* selects only nouns and adjectives to be added as nodes to the graph. After the graph analysis, *PositionRank* considers noun phrases that match the regular expression (adjective)*(noun) + of length up to three to create unigrams, bigrams, and trigrams. It assigns bigrams and trigrams the sum of the scores of its individual unigrams. To create the news background linking search queries using *PositionRank*, and since we only care about weighting single terms, we skip this process of creating bigrams and trigrams, and use the weights assigned by *PositionRank* to the unigrams as is.

#### TopicRank

*TopicRank* is another graph-based keyword extraction algorithm (Bougouin, Boudin & Daille, 2013) that is also based, as *PositionRank*, on the idea of the *PageRank* algorithm. However, in *TopicRank*, the graph nodes are not only single words; instead, a document is represented as a complete graph of topics and the relations between those topics. A topic is defined as a cluster of similar single and multi-word phrases. To define these phrases, *TopicRank* uses a part-of-speech (POS) tagger and extracts the longest sequences of nouns and adjectives from the document as keyphrase candidates. Keyphrase candidates are then split into clusters using a Hierarchical Agglomerative Clustering (HAC) algorithm. After creating the different topics and representing them as nodes, a complete graph is created, where edges are added between topics given their closeness to each other. The weight on the edge between topics is calculated using the reciprocal distances between the offset positions of their keyphrases. The basic *PageRank* on this graph is then applied to weigh the different topics. Finally, *TopicRank* selects the keyphrase from each topic that appears first in the document, and assigns it the topic’s weight. For constructing the search query, we split the extracted keyphrases into unigrams, then we compute the score or boost value of a unigram term *u* as follows: $score(u) = sum_{i = 1}^kTR(i),$ where *k* is the number of the extracted keyphrases that contain the unigram *u*, and $TR(i)$ is the weight assigned by *TopicRank* to an n-gram keyphrase *i*.

#### Multipartite Rank

Based on the idea of creating better representation of keyphrases within the different topics in the a text, Boudin (2018) proposed *Multipartite Rank* (*MPR*). *MPR* uses a more dense graph structure than with *TopicRank*, called multipartite graph to model the topics with their key phrases. In that graph, the nodes are candidate keyphrases that are connected only if they belong to different topics. Accordingly, the document is represented in this graph as tightly connected sets of topic-related candidates/keyphrases. *MPR* also adds an adjustment on the weights of the keyphrases that occur first on each topic. This is based on the assumption that candidates keyphrases that occur at the beginning of the document are better representatives of the topics. We follow the same method as with *TopicRank* to select the required terms and weights.

#### sCAKE

Following the line of graph-based keyword extraction methods, *sCake* (Duari & Bhatnagar, 2019) was motivated by the need to design a paramterless graph construction method. Instead of a sliding window parameter as in *k*-Core or *k*-Truss, in *sCake*, the window slides over two consecutive sentences and the terms co-occurring in these sentences are linked (*i.e*., the edge between a node *n_i_* and node *n_j_* holds how many times the terms *t_i_* and *t_j_* occurred in two consecutive sentences). Similar to *PositionRank*, *sCake* only considers nouns and adjectives as candidate terms for constructing the graph of words. To score nodes in the graph for keyword extraction, *sCake* initially applies the *k*-Truss method explained above, then uses the created truss-hierarchy of subgraphs to assign different features to the nodes, and aggregates those features for each node to compute its final score. These features are the maximum truss number at which an edge between the node and any of its neighbors resides, the sum of truss levels of the node’s neighbors, a positional feature that attempts to favor nodes at the beginning of a document as in *PositionRank*, and a semantic connectivity score that measures the number of distinct concepts that a node links to, assuming that the more the node’s neighbors belong to different concepts, the more important it is. Since *sCake* creates only unigrams, for constructing our search queries, we use the score assigned by *sCake* to each output term as is.

#### Yake

Unlike graph-based methods, *Yake* is a completely statistical method that was introduced recently for keyword extraction (Campos et al., 2020). It depends on capturing five statistical features of terms in a document, and integrating those feature scores in a final score for keyphrase determination. The first feature captures how frequent a term was mentioned starting with a capital letter excluding the beginning of a sentence, or marked as an acronym. This is based on the assumption that uppercase terms are usually more relevant as keywords than lowercase ones. The second feature is term position, which captures, as in *PositionRank*, the position of terms in the document, favoring terms that occur at the beginning of the document. Unlike *PositionRank* though that captures the position of terms itself, *Yake* captures the position of the sentences in which the terms occur. The next feature is a normalized version of the term frequency in a document. The fourth captures the context around the term, based on the assumption that the higher the number of different terms that co-occur with the candidate term on both sides, the less significant term *t* will be. Finally, the last feature captures the percentage of different sentences in which a term occurs, assuming that terms which occur in different sentences are more important. In *Yake*, the smaller the value of a term, the more significant it is. Since in our work we assign boost weights to extracted terms, we assign a score to the term *t* as follows: $score(t) = 1/Yake(t)$.

### Time complexity of keyword extraction techniques

Although we assume an oﬄine extraction of keywords from the query article to construct the search query for background links retrieval, it is worth comparing the time complexity of the aforementioned keyword extraction techniques in case it is performed online, or there is a large number of query articles to be processed by the news provider. Table 2 shows the terminology we used for this analysis, and Table 3 shows the time complexity for each method, along with any specific requirements before the keyword extraction process.

**Table 2:** Symbols used for representing time complexity.

Symbol	Meaning
*N*	Length of the article in words after preprocessing
$n$	Number of unique terms in the article
$s$	Number of sentences in the article
$w$	Width of the sliding window used for constructing the graph of words
$c$	Number of candidate keyphrases determined through POS tagging
$m$	Number of edges in the constructed graph

**Table 3:** Time complexity of keyword extraction algorithms.

Method	Time complexity	Pre-requirements
*Yake*	${{\mathcal{O}}}(N + s\;n\;w)$	Segtok sentence segmenter
*PositionRank*	${{\mathcal{O}}}(N\;{w^2} + c + m)$	POS tagging
*TopicRank*	${{\mathcal{O}}}({c^3} + m)$	POS tagging
*MPR*	${{\mathcal{O}}}({c^3} + m)$	POS tagging
*k*-Core	${{\mathcal{O}}}(N\;{w^2} + m)$	–
*k*-Truss	${{\mathcal{O}}}(N\;{w^2} + n + {m^{1.5}})$	–
*sCake*	${{\mathcal{O}}}(N + s\;{c^2} + {m^{1.5}})$	POS tagging

Most of the graph-based keyword extraction methods are quadratic in time. For instance, constructing a graph of words for *PositionRank*, *k*-Core, or *k*-Truss is of time complexity ${{\mathcal{O}}}((N - w + 1)\;(w(w - 1)/2))$. Since *w* is relatively small with respect to the length of the article, the complexity is ${{\mathcal{O}}}(N{w^2})$. After constructing this graph and assuming $m$ edges in the constructed graph, applying *PositionRank* on the constructed graph additionally requires ${{\mathcal{O}}}(c + m)$ (Vega-Oliveros et al., 2019), as it is a variation of the well known *Pagerank* algorithm with only favoring nodes that appear at the beginning of the article. As for *k*-Core, there is a number of implementations proposed in the literature to decompose the graph of words into cores. The most efficient applies the decomposition in ${{\mathcal{O}}}(m)$ time (Batagelj & Zaversnik, 2003). While *k*-Truss is relatively more complex than *k*-Core when computed on large graphs, an in memory-based complexity of ${{\mathcal{O}}}({m^{1.5}})$ can be considered (Wang & Cheng, 2012), which suits the relatively small graph of words for news articles.

As for *TopicRank* and *MPR*, both need POS tagging before constructing the text graph. Although the number of candidate keyphrases $c$, obtained after POS tagging, is less than the number of unique terms $n$, both algorithms apply agglomerative clustering to the candidate keyphrases, which is of worst time complexity ${{\mathcal{O}}}({c^3})$. The construction of the topic graph then requires ${{\mathcal{O}}}(N.c)$ for computing the candidate keyphrase offset positions, and ${{\mathcal{O}}}({c^2})$ for computing the positional distance between pairs of candidate keyphrases. Both algorithms additionally require ${{\mathcal{O}}}(c + m)$ for applying *PageRank*.

For *sCake*, aside also from the need to apply POS tagging, it requires ${{\mathcal{O}}}(N)$ to calculate term frequencies, ${{\mathcal{O}}}({c^2}*s)$ to construct the graph, ${{\mathcal{O}}}({m^{1.5}})$ for *k*-Truss, and for computing the positional weights.

Finally, *Yake* depends (as mentioned in Campos et al. (2020)) on the rule-based segtok algorithm (https://pypi.org/project/segtok/) that segments a text into sentences based on orthographic features, and tokenizes the text into terms with delimiter tags (*e.g*., Acronyms, Uppercase, Digits, *etc*.). After identifying the sentences, *Yake* takes ${{\mathcal{O}}}(N)$ for computing the term frequencies and casing features. It additionally requires ${{\mathcal{O}}}(s.n.w)$ for context feature calculations. We omit here the complexity to generate n-gram keyphrases as our goal is to obtain unigram terms. Hence, compared to the methods mentioned above, it is considered the most efficient method for extracting unigram terms.

As for *TF*, the time complexity is ${{\mathcal{O}}}(N)$ with no additional requirements, making it the most efficient method among the others. For *TF*-*IDF*, assuming a constant time ${{\big (\mathcal{O}}}(1)\big)$ to get the document frequency of each term from the index, the complexity remians ${{\mathcal{O}}}(N)$, making it similarly efficient as *TF*.

## Experimental evaluvation

In this section, we present our experimental evaluation. We first describe our experimental setup, followed by a discussion on the experimental results that address our research questions.

### Experimental setup

Our experimental setup covers the dataset description, the pre-processing and indexing phase, the baseline method, some implementation issues, the retrieval method, and the evaluation measures.

#### Dataset

We conducted our experiments on version 3 of the Washington Post news test collection released by TREC for the background linking task in the news track (https://trec.nist.gov/data/wapost/). The collection covers 7 years of articles (2012–2019) with about 672 k documents comprising news articles, columns, and blogs. It also contains 50, 57, and 49 query articles released in 2018, 2019, and 2020 respectively. Each of those query articles is associated with a set of background articles manually-judged on a 0–4 relevance scale indicating how much context and background knowledge they provide to the query article (Soboroff, Huang & Harman, 2018, 2019, 2020).

#### Preprocessing and indexing

Each article in the dataset is formatted as a JSON object, which is broken into a title and multiple content paragraphs. For each article, we extracted the metadata (title, author URL, and publishing date) and concatenated the text content (marked by a “sanitized_html” type). We used JSOUP library (https://jsoup.org/) to extract the raw text from the HTML text. Afterwards, we lower-cased the text and removed stop words. Finally, the pre-processed text was indexed, along with the article’s meta-data, using Lucene v8 (http://lucene.apache.org/). We did not perform stemming as, per preliminary experiments with the full-article baseline, the performance was degraded compared to non-stemming.

#### Baseline

Since the news background linking problem was mainly and extensively addressed within the scope of TREC conference, we elected to choose the baseline as the most (to date) effective method as reported by TREC (Soboroff, Huang & Harman, 2018, 2019, 2020). It uses the concatenated text of the query article’s title and its body content (after stop words removal) as a search query to retrieve the background links. While there were few attempts outside TREC to address this problem, none were able to achieve a better performance than this simple baseline method, constituting the state of the art on that problem. Accordingly, we focus our comparison with only this baseline, aiming at achieving the same effectiveness, but with much shorter, hence efficient, search queries.

#### Implementation issues

Due to lack of open source implementation, we implemented our *k*-Core and *k*-Truss keyword extraction methods in Java. For *PositionRank* (https://github.com/ymym3412/position-rank), *Yake* (https://github.com/LIAAD/yake), and *sCake* (https://github.com/SDuari/sCAKE-and-LAKE), we used the authors’-provided implementation developed in Python for the first two methods and *R* for the third. For *TopicRank* and *MPR*, we used the PKE Python library (https://github.com/boudinfl/pke). We set the sliding window to three for methods that required it (*i.e*., *PositionRank*, *k*-Core, and *k*-Truss). For *Yake*, we set the maximum n-gram size to one to get only unigram keywords, and the window size to 1, as recommended in Campos et al. (2020). For the other methods, we used the default parameters settings as suggested by their given libraries. For instance, for *MPR*, we set alpha to 1.1, and the threshold to 0.74. For *PositionRank*, we set alpha to 0.85.

#### Retrieval

After keyword extraction, *i.e*., generating the search query, the constructed index was used to retrieve the set of candidate background articles. We used the default Lucene scoring function, which is an implementation of the BM25 retrieval model, to score the articles in all of our experiments^1^ 1We experimented with other retrieval models implemented within Lucene, however, the default one achieved the best results for the baseline method, therefore, we adopted it for all other methods.. Upon retrieval of the candidate articles, we filtered the results to exclude articles that are “Opinions”, “Letters to the Editor”, or “The Post’s View” as they were declared by TREC to be non-relevant. We also excluded articles that were published after the query article.

#### Evaluation measures

We used nDCG@5 as our primary evaluation measure for effectiveness, since it was used as the primary measure by TREC. The gain value for each retrieved background article was calculated as *2^r^*, where *r* indicates the relevance level between 0 (non relevant) and 4 (the most relevant). For reliable evaluation, we evaluated the studied methods using 3-fold cross validation over the TREC query articles. For easier future comparisons, the folds were chosen to be the query sets released by TREC in each year for the background linking task (2018, 2019 and 2020), *i.e*., two TREC query sets of two years were used for parameter tuning, and the third was used for testing. The only parameter we tuned in our experiments was the number of extracted terms that are used to construct the search query.

To report efficiency, we use the query response time in milliseconds. We ran all our experiments on a MacBook Pro machine with a 2.5 GHz Quad-Core Intel Core i7 processor, and 16 GB 1600 MHz DDR3 memory. The reported time per query is the average time of three runs of the same experiment.

### Experimental results

In this section, we discuss in detail the different experiments that we conducted to address our research questions. We first test the effectiveness of the simple lead-paragraphs extraction method. Then, we discuss the effectiveness and efficiency of the queries generated by the different keyword extraction methods for our task. Finally, we show the potential of each of the keyword extraction methods with *post hoc* reranking of the retrieved articles to reach better retrieval effectiveness.

#### Leading paragraphs as search queries (RQ1)

When authors write news articles, they often adopt the inverted pyramid style (DeAngelo & Yegiyan, 2019; Pottker, 2003), in which the essential and most attention-grabbing elements are introduced *first* in the article. Accordingly, in this section we address **RQ1**, that is to test if the lead paragraphs of the articles can simply and sufficiently be used as a search query instead of the whole article for the retrieval of the background links. In other words, we wanted to check if applying keyword extraction is at all required.

**Figure 4:**
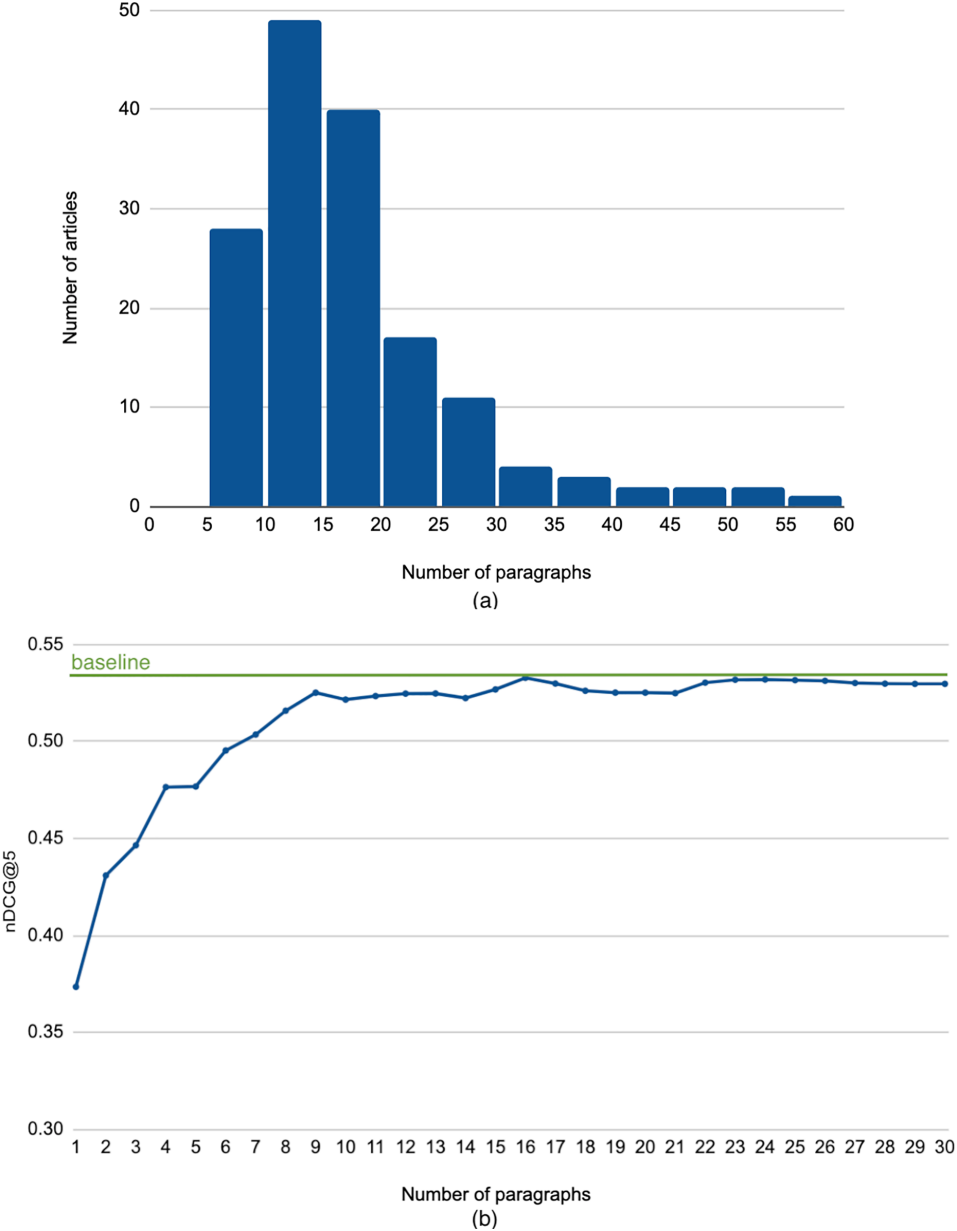
(A) Histogram of the length of the query articles. (B) Performance using leading paragraphs as search query.

To answer this question, we experimented with constructing search queries simply using the top paragraphs of the query article, after stop words removal. Figure 4A shows the histogram of the length of the query articles in paragraphs^2^ 2Only one very long query article (141 paragraphs) was omitted from the histogram for clarity.. The figure indicates that most articles have less than 20 paragraphs, with an average of about 18 paragraphs. Figure 4B illustrates the performance when we vary the number of top paragraphs from 1 (just the first paragraph of the article) up to 30 (almost the full article). We observe that increasing the number of paragraphs used for constructing the search query improves the performance. We also note that the peak nDCG@5 occurred at using 16 paragraphs, which is very close to the average number of paragraphs. This indicates that using only a few lead paragraphs is not sufficient for an effective background links retrieval.

**Figure 5:**
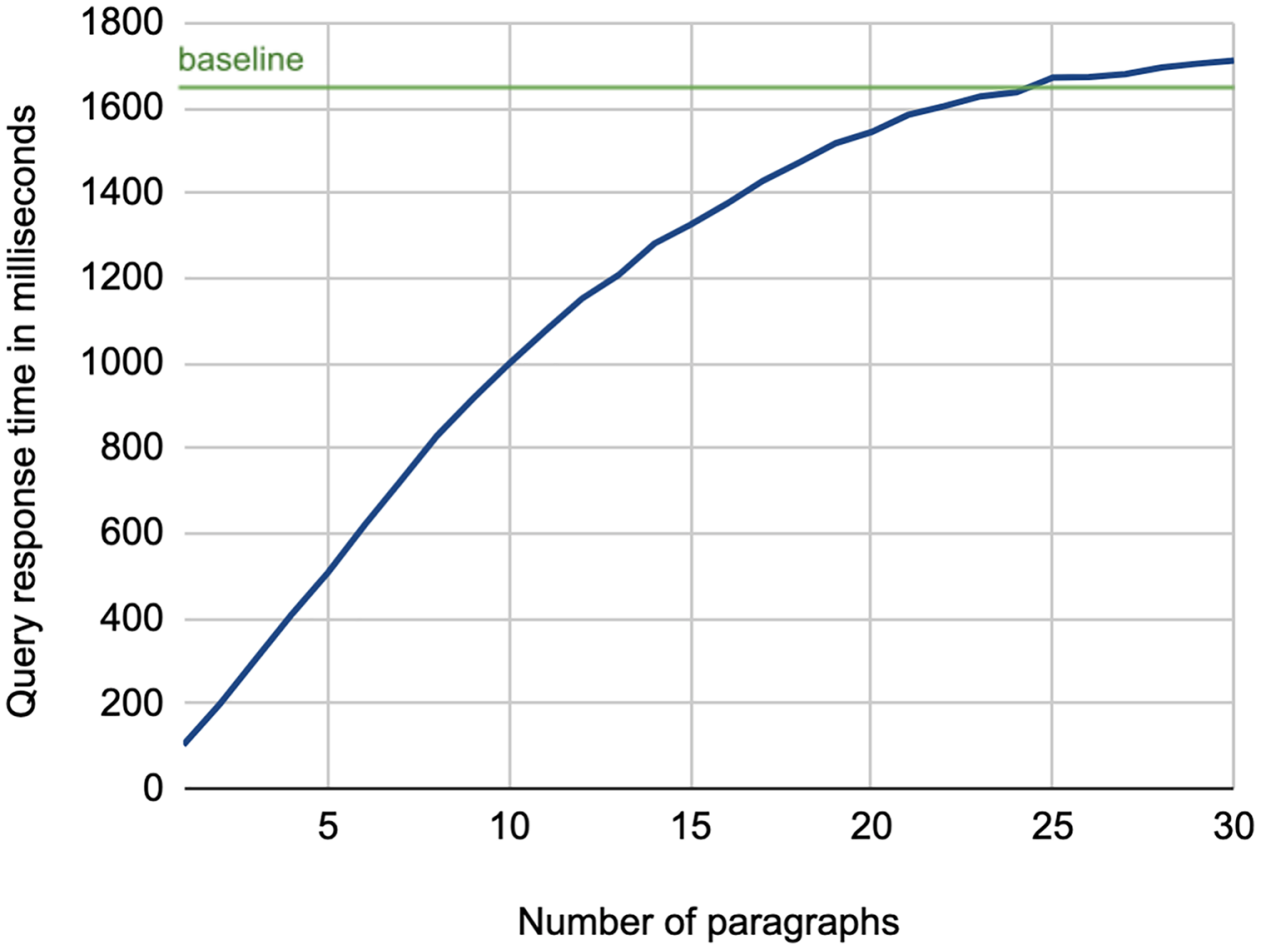
Average query response time for retrieving background links using lead paragraphs as a search query.

As for the efficiency of the retrieval process, Fig. 5 shows the average query response time using the lead paragraphs as search queries. As illustrated, there is a high growth in response time while increasing the number of paragraphs, which increases the number of terms in the search query^3^ 3There were two outlier queries that took more time compared to others for processing. However, removing both did not noticeably affect the average processing time.. This experiment confirms the need for a keyword extraction mechanism that can select few terms to construct the search query while maintaining the effectiveness of using the full article.

While we might expect that the baseline method should always exhibit the highest response time, the figure shows it is not the case when using 25 to 30 paragraphs. This is due to the optimization of query processing performed by the Lucene platform, which adopts *BlockMax WAND* approach that skips scoring some documents in the posting lists of terms when their score contribution is expected to be not competitive (hence not affecting the document ranking), resulting in a faster retrieval process (Grand et al., 2020). This indeed is more evident in long queries, as in the baseline case.

#### Keyword extraction for search query reduction (RQ2)

**Figure 6:**
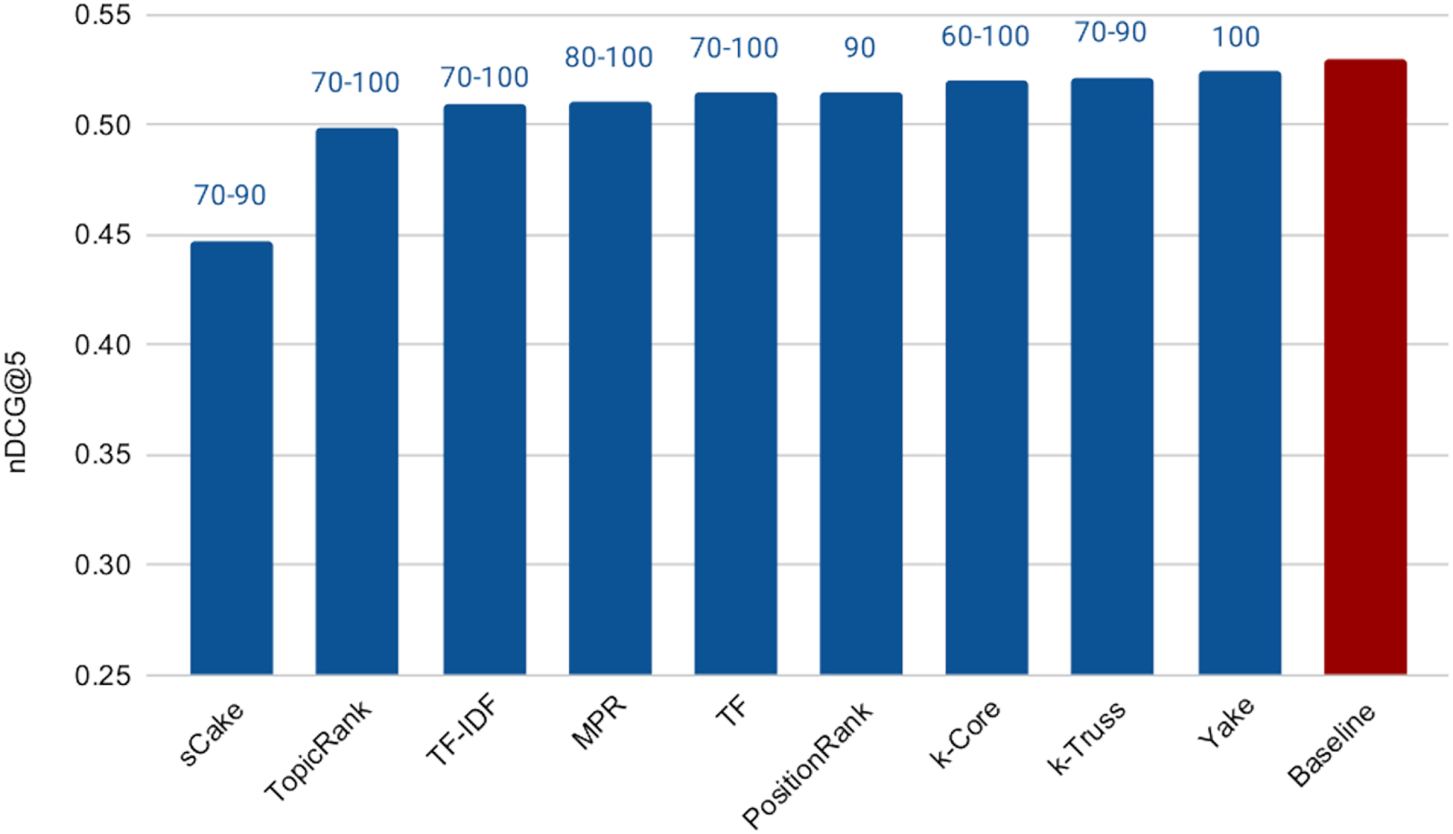
Effectiveness of keyword extraction techniques varying the number of extracted keywords from 30 to 100.

The next experiment aims at addressing **RQ2**, which is concerned with measuring the effectiveness of the keyword extraction techniques in search query reduction for background linking. To conduct this experiment, we applied each keyword extraction method explained earlier to analyze the query articles and construct search queries. Figure 6 shows the cross-validation results varying the number of extracted keywords from 30 (since our preliminary experiments in this regard showed that the effectiveness degrades noticeably if the number of search terms are less than 30) to 100 (for efficiency purposes). It also reports the performance of the *Baseline* method. The range of the optimal (after tuning) values of the number of extracted keywords (over the three folds) for each method is reported on top of the respective bar. Surprisingly, all methods, except *sCake*, exhibit very similar performance with small differences in effectiveness. Accordingly, we applied the paired two-sample t-test with 5% significance level between the baseline and each of the other methods; we found that the differences in performance between the baseline and each of *Yake*, *k*-Truss, *k*-Core, *PositionRank*, and *MPR* methods are *not* statistically significant, which means that those five methods essentially exhibit the same effectiveness of the baseline despite using much shorter queries. This, in turn, achieves our goal of having no sacrifice in the retrieval effectiveness as a result of the query reduction process.

**Figure 7:**
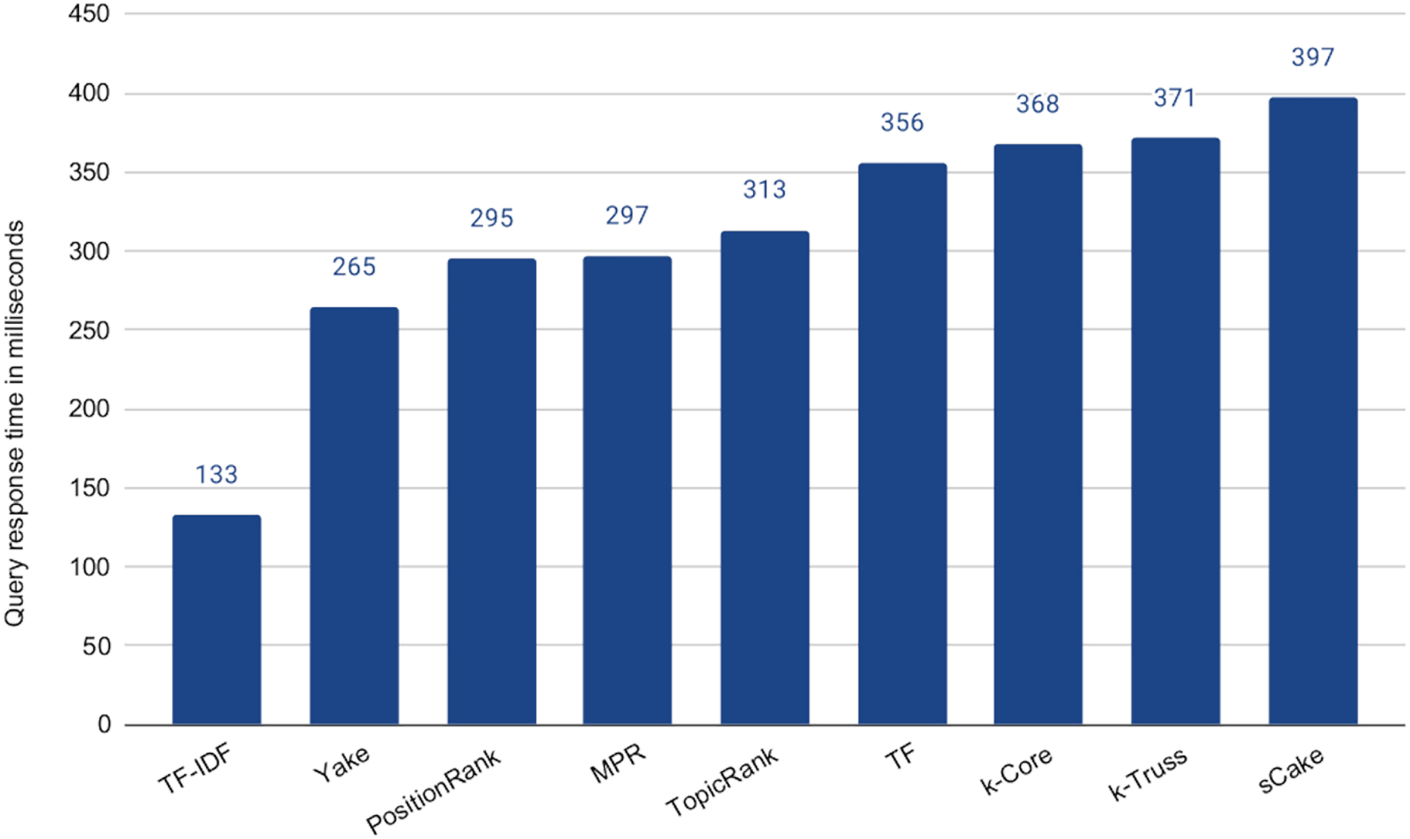
Average query response time for 100-term search queries generated by the keyword extraction methods.

Being effectively similar, using any of the methods that exhibit no statistical significant difference with the baseline is expected to be more efficient than the baseline while maintaining the retrieval effectiveness. This is due to the much shorter search queries. Recall that the average length of the query articles is 400 terms *vs* maximum of 100 terms in the above experiment, *i.e*., 75% reduction in the query length. However the selection of the query terms might impact the speedup. To check that, we computed the average query response time for each of the keyword extraction methods for 100-term queries, as shown in Fig. 7. In this experiment, we did not tune the number of terms, as we choose to test the worst case scenario, which is using our ceiling number of terms (*i.e*., 100). It is then intuitive that the time taken for processing a subset of those terms will be lower. Among the five methods above that exhibit the indifferent performance with the baseline, *Yake* is the fastest with about **6.2×** speedup over the baseline (which takes 1,645 ms, but omitted from the figure due to scale). Recall that *Yake* is also the closest in nDCG@5 performance to the baseline, among all other methods (as illustrated in Fig. 6), and also the fastest (after the two *TF*-based methods) in the keyword extraction process (as shown in the time complexity analysis earlier). It indeed yields a perfect solution for the practical scenarios. We also notice that the queries generated by the *TF*-*IDF* method are the most efficient ones. This is expected as the method prioritizes terms of high IDF, *i.e*., short postings lists. Therefore, if we need even higher speedup (**12.4×** over the baseline in this case) while tolerating some degradation in effectiveness, *TF*-*IDF* is then the choice.

**Figure 8:**
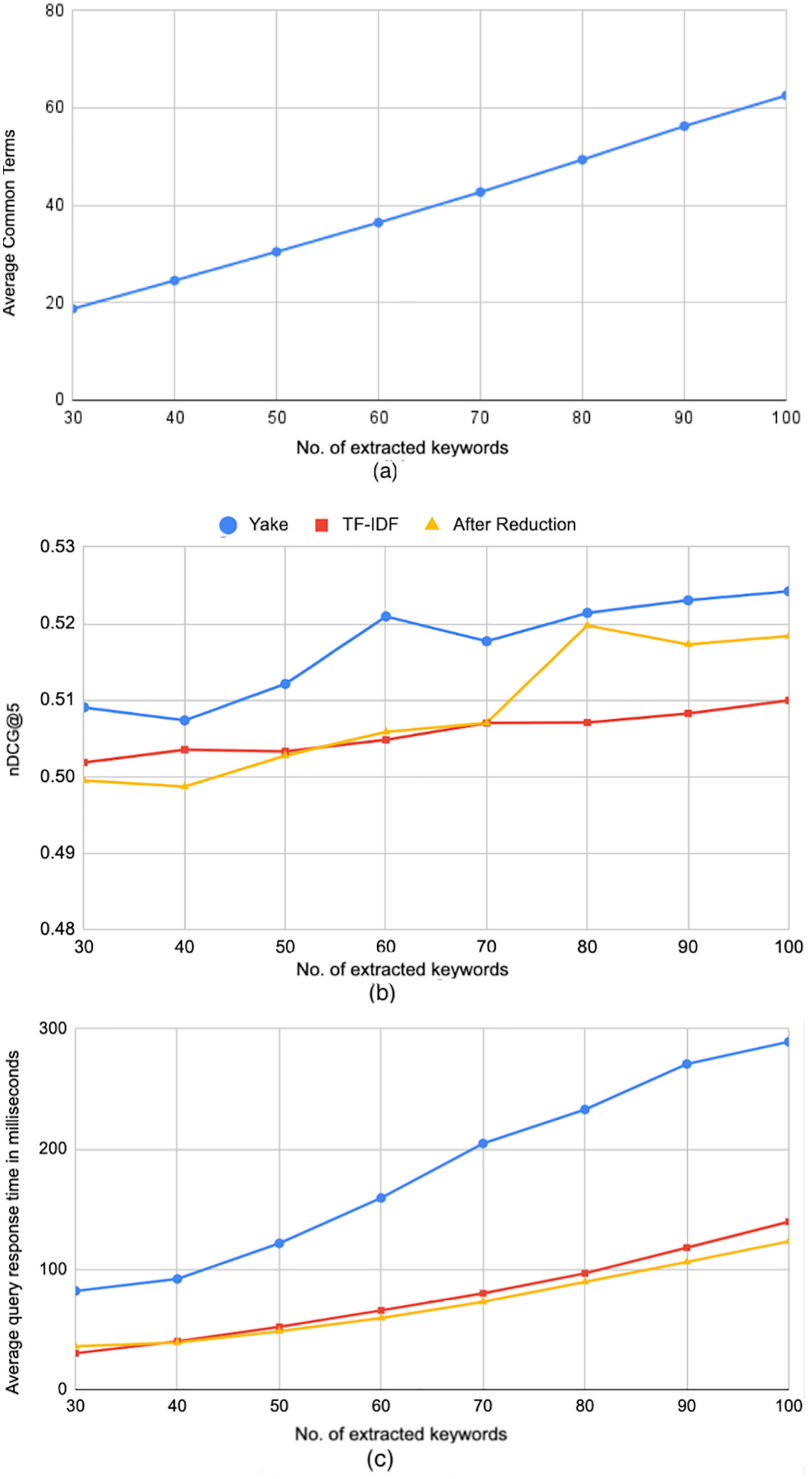
Further reduction of search query terms. (A) The average query length after keeping only the common extracted terms by *TF-IDF* and *Yake* methods. (B) Effectiveness of *Yake* and *TF-IDF* methods before reduction, and *Yake* after reduction. (C) The average processing time of queries after applying the reduction.

The results above inspired us to check if the terms in the queries generated by *Yake* are different than the ones generated by the *TF*-*IDF* method (hence the difference in the processing time), and if those different terms actually influence the retrieval effectiveness. To answer this question, we computed the average cosine similarity between the search query vectors generated by both methods over queries of different lengths. The resulted average similarity was 0.8, indicating that *TF*-*IDF* yields somewhat different queries. That motivated us to conduct another exploratory followup experiment, where we only keep the terms that are commonly extracted by *TF*-*IDF* and *Yake*. Figure 8A shows the average length of the queries after this filtering for different number of originally extracted keywords. The figure shows considerable reduction; for example, 27 terms were filtered out on average when the original queries have 100 terms, keeping only 63 terms in common. As for the effectiveness past the reduction of the queries, it can be seen in Fig. 8B that when the originally-extracted terms exceeds 80, the reduced queries yield very close effectiveness, in terms of nDCG@5, to the original queries generated by *Yake*. In fact, the difference in performance between the baseline and the query reduced from 100 extracted terms is *not* statistically significant, indicating that the removed terms were quite ineffective, yielding a more efficient retrieval. This can be clearly noticed in Fig. 8C, which shows that the reduced queries are much more efficient than the queries generated by *Yake*, and a bit more efficient than the queries generated by *TF*-*IDF*, yielding about 13.3× average speedup over the baseline.

In conclusion, several keyword extraction methods exhibit similar effectiveness (with no statistical significance difference) to the baseline while being much more efficient. Among those, the queries generated by *Yake*, in particular, yield 6.2× speedup over the baseline. Moreover, *Yake*’s queries can be further reduced by filtering out terms that were not selected by *TF*-*IDF* method, yielding even better average of 13.3× speedup in response time over the baseline, with a little cost of running both (already fast) methods of keyword extraction. Again, the difference in effectiveness in that case is not statistically significant. It is important to note here that while the reported reduction in the response time is in terms of milliseconds in this in-lab experiment, such speedup is highly and typically needed during Web search tasks (such as searching for background sources over the Web) in real-time online scenarios.

#### How far can we reach with reranking? (RQ3)

We observe that the most effective method reported for background linking, including the baseline method, is still far from being optimal (maximum reached effectiveness is nDCG@5 = 0.53). But what if a method retrieves relevant background links at lower ranks than top 5? Those links will not be considered when computing the nDCG@5 score (*i.e*., will not be shown to the user). However, this initial set of retrieved links can be re-ranked by another downstream method (*e.g*., by fine-tuning pre-trained models) to further improve the effectiveness. To measure how far any of the keyword extraction methods can potentially reach in terms of nDCG@5 with *post hoc* reranking, we retrieve, using each method, a set of *N* candidate articles, then we optimally rerank those articles using the judgement scores assigned by TREC annotators. Not-annotated articles are considered non-relevant, and thus are assigned a 0 score. Doing so, we obtain oracle runs for the different methods. We then compute nDCG@5 for those runs.

**Figure 9:**
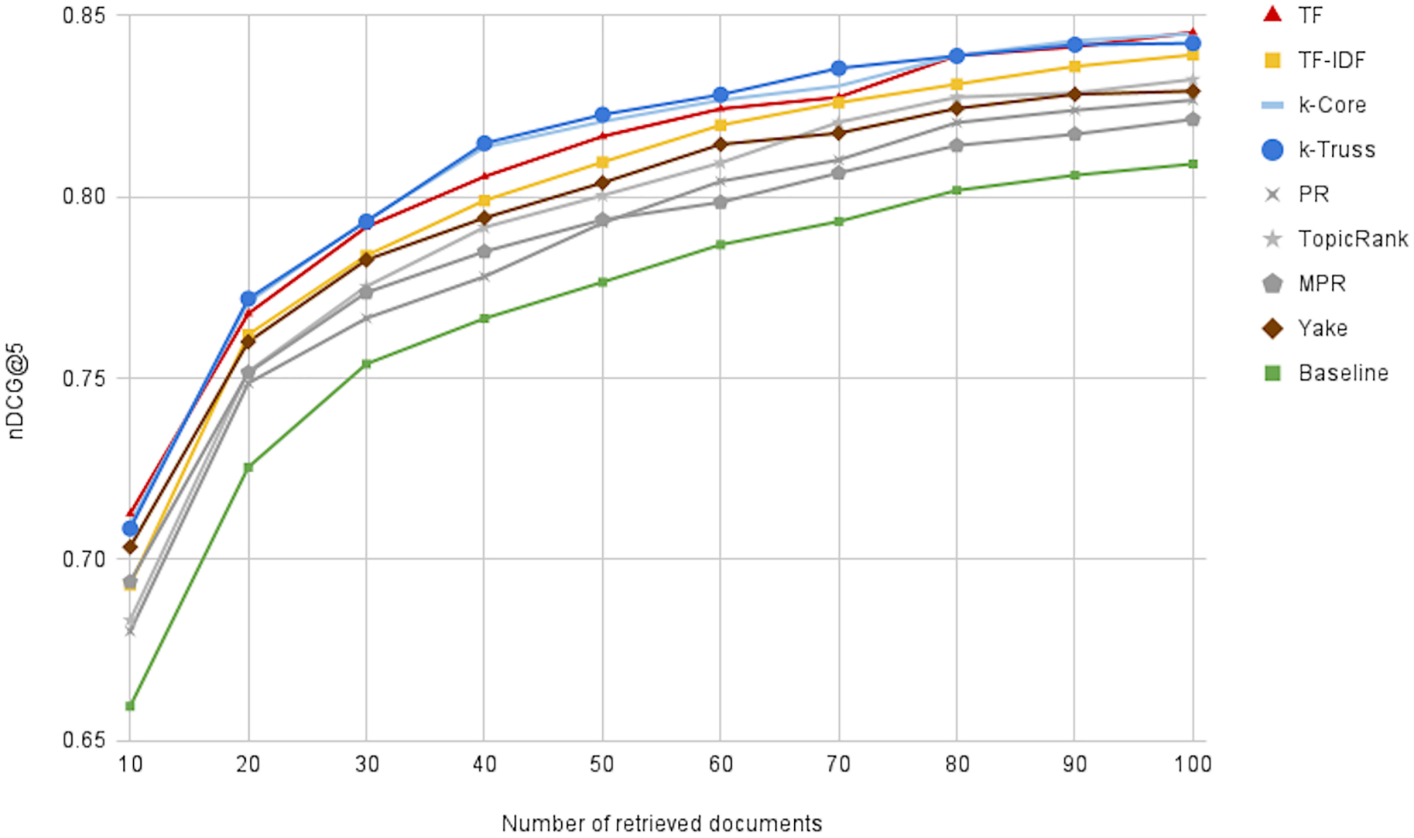
The effectiveness of the oracle runs generated by optimally reranking the top *N* retrieved documents.

Figure 9 illustrates the performance of the oracle runs, changing *N* from 10 to 100, for all keyword extraction methods, besides the baseline method. We first notice that the performance can reach 0.84 at *N* = 100, indicating the great potential for reranking the top 100 retrieved articles. Moreover, we can notice that the *k*-Truss and *k*-Core methods exhibit the best performance (*i.e*. obtaining the most promising lists), followed by *TF* and *TF*-*IDF*. That suggests another criterion of selecting a keyword extraction technique for our task, which is the potential for better effectiveness with postdoc reranking. We also note that the baseline method has the *lowest* reranking potential, indicating that keyword extraction is not only needed for efficiency purposes, but also needed for increasing the potential of having more relevant background articles at the top of the retrieved list.

## Conclusion and future work

News background linking is still an open research problem that was introduced recently to the research community. The most effective method proposed to date addresses this problem as an *ad-hoc* retrieval one, where the entire input news article is used as a search query to retrieve the background links from an indexed collection. In a scenario where the lookup for background links is performed online, this method becomes inefficient, especially if the search scope is big, such as the Web. In this article, we proposed to reduce the news article to much fewer terms to efficiently retrieve the background links, while maintaining the same effectiveness of the state-of-the-art method. For search query reduction, we studied several unsupervised keyword extraction techniques, along with standard baseline ones. Our results showed that we reach large speedup in query response time using simple statistical keyword extraction techniques, such as *Yake*, with a difference in effectiveness that is not statistically significant from the state-of-the-art method. We further showed that by adopting *TF*-*IDF* traditional keyword extraction method to filter non-informative terms from the queries generated by *Yake*, one may further achieve even higher speedup, still with similar effectiveness. We also showed there is a huge potential in further reranking the candidate background links to reach higher effectiveness using graph-based keyword extraction techniques, such as *k*-Truss and *k*-Core, for forming the initial search query that exhibit similar effectiveness with the full-article search approach, yet they are much more efficient. Since the most reported effectiveness for the news background linking problem is still far from being optimal, our future work will focus on investigating how to link articles through methods that go beyond lexical similarity. More specifically, we plan to experiment with transfer learning techniques to build a system that can rerank an initial retrieved set of background links. Transformer-based pretrained models that leverage semantic similarity of short or long documents are clear candidate learning-to-rank models for that task.

## Additional Information and Declarations

### Data Availability

The following information was supplied regarding data availability: The raw data is available at Zenodo: Marwa-Essam81. (2022). Marwa-Essam81/EfficientNewsBL: @newsbackroundLinking (@newsbackgroundlinking). Zenodo. https://doi.org/10.5281/zenodo.7329399.

## References

[ref-1] Ajnadkar O, Jaiswal A, Gourav Sharma P, Shekhar C, Soren AK, Mandal JK, Mukherjee I, Bakshi S, Chatterji S, Sa PK (2021). News background linking using document similarity techniques. Computational Intelligence and Machine Learning.

[ref-2] Ak AE, Köksal Ç, Fayoumi K, Yeniterzi R (2020). SU-NLP at TREC news 2020.

[ref-3] Bae J, Kim S (2014). Identifying and ranking influential spreaders in complex networks by neighborhood coreness. Physica A: Statistical Mechanics and its Applications.

[ref-4] Balasubramanian N, Kumaran G, Carvalho VR (2010). Exploring reductions for long web queries.

[ref-5] Batagelj V, Zaversnik M (2003). An O(m) algorithm for cores decomposition of networks. CoRR.

[ref-6] Bimantara A, Blau M, Engelhardt K, Gerwert J, Gottschalk T, Lukosz P, Piri S, Shaft NS, Berberich K (2018). htw saar @ TREC 2018 news track.

[ref-7] Bisandu DB, Prasad R, Liman MM (2018). Clustering news articles using efficient similarity measure and N-grams. International Journal of Knowledge Engineering and Data Mining.

[ref-8] Boudin F, Walker MA, Ji H, Stent A (2018). Unsupervised keyphrase extraction with multipartite graphs. Proceedings of the 2018 Conference of the North American Chapter of the Association for Computational Linguistics: Human Language Technologies.

[ref-9] Bougouin A, Boudin F, Daille B (2013). TopicRank: graph-based topic ranking for keyphrase extraction.

[ref-10] Campos R, Mangaravite V, Pasquali A, Jorge A, Nunes C, Jatowt A (2020). Yake! Keyword extraction from single documents using multiple local features. Information Sciences.

[ref-12] Chakraborty A, Ganguly D, Caputo A, Jones GJ (2022). Kernel density estimation based factored relevance model for multi-contextual point-of-interest recommendation. Information Retrieval Journal.

[ref-13] Chakraborty A, Ghosh S, Ganguly N, Gummadi KP (2019). Optimizing the recency-relevance-diversity trade-offs in non-personalized news recommendations. Information Retrieval Journal.

[ref-14] Cohen J (2008). Trusses: cohesive subgraphs for social network analysis.

[ref-15] Conrad JG, Bender M (2016). Semi-supervised events clustering in news retrieval.

[ref-16] Daiber J, Jakob M, Hokamp C, Mendes PN (2013). Improving efficiency and accuracy in multilingual entity extraction.

[ref-17] Day N, Worley D, Allison T (2020). OSC at TREC 2020-news track’s background linking task.

[ref-11] DeAngelo TI, Yegiyan NS (2019). Looking for efficiency: how online news structure and emotional tone influence processing time and memory. Journalism & Mass Communication Quarterly.

[ref-18] Deshmukh AA, Sethi U (2020). IR-BERT: leveraging BERT for semantic search in background linking for news articles. ArXiv preprint.

[ref-19] Ding Y, Lian X, Zhou H, Liu Z, Ding H, Hou Z (2019). ICTNET at TREC 2019 news track.

[ref-20] Duari S, Bhatnagar V (2019). sCAKE: semantic connectivity aware keyword extraction. Information Sciences.

[ref-21] Essam M, Elsayed T (2019). bigIR at TREC 2019: graph-based analysis for news background linking.

[ref-22] Essam M, Elsayed T (2020). Why is that a background article: a qualitative analysis of relevance for news background linking.

[ref-23] Florescu C, Caragea C (2017). PositionRank: an unsupervised approach to keyphrase extraction from scholarly documents.

[ref-24] Foley J, Montoly A, Pena M (2019). Smith at TREC 2019: learning to rank background articles with poetry categories and keyphrase extraction.

[ref-25] Gautam R, Mandar Mitra DR (2020). TREC 2020 news track background linking task.

[ref-26] Grand A, Muir R, Ferenczi J, Lin J (2020). From MaxScore to block-max wand: the story of how lucene significantly improved query evaluation performance.

[ref-27] Hu L, Li C, Shi C, Yang C, Shao C (2020). Graph neural news recommendation with long-term and short-term interest modeling. Information Processing & Management.

[ref-28] Khan M, Rahman AU, Ullah M, Naseem R (2018). The role of named entities in linking news articles during preservation.

[ref-29] Khloponin P, Kosseim L (2019). The CLaC system at the TREC 2019 news track.

[ref-30] Khloponin P, Kosseim L (2021). Using document embeddings for background linking of news articles.

[ref-31] Kumaran G, Carvalho VR (2009). Reducing long queries using query quality predictors.

[ref-32] Le Q, Mikolov T (2014). Distributed representations of sentences and documents.

[ref-33] Lehmann J, Castillo C, Lalmas M, Baeza-Yates R (2017). Story-focused reading in online news and its potential for user engagement. Journal of the Association for Information Science and Technology.

[ref-34] Lin AY, Ford J, Adar E, Hecht B (2018). VizByWiki: mining data visualizations from the web to enrich news articles.

[ref-35] Lopez-Ubeda P, Diaz-Galiano MC, Valdivia MTM, Urena-Lopez LA (2018). Using clustering to filter results of an information retrieval system.

[ref-36] Lu K, Fang H (2019). Leveraging entities in background document retrieval for news articles.

[ref-37] Lua K, Fang H (2018). Paragraph as lead—finding background documents for news articles.

[ref-38] Macdonald C, Tonellotto N, Ounis I (2012). Learning to predict response times for online query scheduling.

[ref-39] Metzler D, Croft WB (2007). Linear feature-based models for information retrieval. Information Retrieval Journal.

[ref-40] Miah M, Sulaiman J, Sarwar TB, Zamli KZ, Jose R (2021). Study of keyword extraction techniques for electric double-layer capacitor domain using text similarity indexes: an experimental analysis. Complexity.

[ref-41] Mihalcea R, Tarau P (2004). TextRank: Bringing order into text.

[ref-42] Missaoui S, MacFarlane A, Makri S, Gutierrez-Lopez M (2019). DMINR at TREC news track.

[ref-43] Moffat A, Webber W, Zobel J, Baeza-Yates R (2007). A pipelined architecture for distributed text query evaluation. Information Retrieval Journal.

[ref-44] Naskar A, Saha R, Dasgupta T, Dey L (2019). Ontology guided purposive news retrieval and presentation.

[ref-45] Nicholls T, Bright J (2019). Understanding news story chains using information retrieval and network clustering techniques. Communication Methods and Measures.

[ref-46] O’Brien HL (2011). Exploring user engagement in online news interactions. Proceedings of the American Society for Information Science and Technology.

[ref-70] Örs FK, Yeniterzi S, Yeniterzi R (2020). Event clustering within news articles.

[ref-47] Page L, Brin S, Motwani R, Winograd T (1999). The pagerank citation ranking: bringing order to the web.

[ref-48] Papagiannopoulou E, Tsoumakas G (2020). A review of keyphrase extraction. Wiley Interdisciplinary Reviews: Data Mining and Knowledge Discovery.

[ref-49] Phan MC, Sun A (2018). CoNEREL: collective information extraction in news articles.

[ref-50] Piskorski J, Stefanovitch N, Jacquet G, Podavini A (2021). Exploring linguistically-lightweight keyword extraction techniques for indexing news articles in a multilingual set-up.

[ref-51] Pottker H (2003). News and its communicative quality: the inverted pyramid—when and why did it appear?. Journalism Studies.

[ref-52] Qian Y, Deng X, Ye Q, Ma B, Yuan H (2019). On detecting business event from the headlines and leads of massive online news articles. Information Processing & Management.

[ref-53] Qiu Y, Frei H-P (1993). Concept based query expansion.

[ref-54] Qu J, Wang Y (2019). UNC SILS at TREC 2019 news track.

[ref-55] Rabby G, Azad S, Mahmud M, Zamli KZ, Rahman MM (2020). TeKET: a tree-based unsupervised keyphrase extraction technique. Cognitive Computation.

[ref-56] Ravenscroft J, Clare A, Liakata M (2018). HarriGT: linking news articles to scientific literature.

[ref-57] Rousseau F, Vazirgiannis M (2015). Main core retention on graph-of-words for single-document keyword extraction.

[ref-58] Rudnik C, Ehrhart T, Ferret O, Teyssou D, Troncy R, Tannier X (2019). Searching news articles using an event knowledge graph leveraged by wikidata.

[ref-59] Salih NM, Jacksi K (2020). State of the art document clustering algorithms based on semantic similarity. Jurnal Informatika.

[ref-60] Sarwar TB, Noor NM, Miah MSU (2022). Evaluating keyphrase extraction algorithms for finding similar news articles using lexical similarity calculation and semantic relatedness measurement by word embedding. PeerJ Computer Science.

[ref-61] Soboroff I, Huang S, Harman D (2018). TREC 2018 news track overview.

[ref-62] Soboroff I, Huang S, Harman D (2019). TREC 2019 news track overview.

[ref-63] Soboroff I, Huang S, Harman D (2020). TREC 2020 news track overview.

[ref-64] Tixier A, Malliaros F, Vazirgiannis M (2016). A graph degeneracy-based approach to keyword extraction.

[ref-65] Vega-Oliveros DA, Gomes PS, Milios EE, Berton L (2019). A multi-centrality index for graph-based keyword extraction. Information Processing & Management.

[ref-66] Wang J, Cheng J (2012). Truss decomposition in massive networks. Proceedings of the VLDB Endowment.

[ref-67] Weichselbraun A, Kuntschik P, Braşoveanu AM (2018). Mining and leveraging background knowledge for improving named entity linking.

[ref-68] Yang P, Fang H, Lin J (2017). Anserini: enabling the use of lucene for information retrieval research.

[ref-69] Yang P, Lin J (2018). Anserini at TREC 2018: centre, common core, and news tracks.

